# Trans-prosthetic recanalization of a collapsed iliac limb using the GoBack catheter: a case report

**DOI:** 10.1186/s42155-021-00244-4

**Published:** 2021-07-07

**Authors:** Andrea Azzaretti, Daniele Trevisan, Camilla Fachinetti, Claudia Borghi, Alberto Vannelli

**Affiliations:** 1 FF UOC Radiodiagnostica, Valduce Hospital, Via Dante Alighieri 11, 22100 Como, Italy; 2 U.O.C. Chirurgia Generale, Valduce Hospital, Via Dante Alighieri, 11, 22100 Como, Italy

**Keywords:** Graft limb occlusion, Aortoiliac occlusion, Crossing device, Peripheral angioplasty

## Abstract

**Background:**

Recanalization of graft limb occlusion can prove challenging and the use of the GoBack crossing and reentry device may be a suitable option, especially when there is no other way to restore flow with an usual endovascular approach. The GoBack catheter is a novel device designed to enhance pushability, and to enable direction-change inside hard plaques and crossing of tough lesions, even when they involve graft fabric.

**Case presentation:**

It’s reported a case of a 76-year-old male who presented with claudication, previous placement of an aorto-bi iliac graft by open surgery for a ruptured abdominal aneurysm 10 years ago that, over time, developed severe kinking on the left limb and a fabric occlusion on the right limb. After several unsuccessful attempts to cross the occlusion of the right common iliac artery, the GoBack™ was deployed to create a lumen through graft’s folds. After angioplasty and stenting a satisfactory result was achieved, restoring flowCT-scan at 1 month and duplex ultrasound (DUS) at 3 months confirmed the patency of ilio-femoral axis.

**Conclusions:**

The advent of this new CTO crossing device has the potential to facilitate recanalization of some of the most challenging occlusions. Facilitating more consistent distal entry and allowing for a decrease in crossing time. Therefore, the GoBack catheter should be considered as a potential complementary tool to treat vascular occlusions via endovascular approaches, especially when classical endovascular techniques fail.

## Background

Management of graft limb occlusion is relatively straightforward and can usually be accomplished via endovascular techniques. In this endovascular era, however, the ability to treat lesions is limited primarily by failure to cross the occlusion that often leads the surgical approach to be the only suitable option. Regarding to endovascular treatments of occlusions, a considerable impact of the procedural success rate was related to improved wire technology, wire strategies and the development of devices (Schneider et al. 2013; Rogers and Laird 2007), to solve complex and potentially failed intraluminal attempts as well as very particular cases as this here reported, where the multifunctionality of this novel device, GoBack™, helped to overcome a difficult situation and avoided alternative approaches to the endovascular one. The GoBack™ Crossing Catheter (Upstream Peripheral Technologies, Caesaria - Israel) is a single-lumen crossing catheter which features a curved nitinol needle at the distal tip, with an adjustable protrusion length from a straight (2–3 mm) to a fully curved (11 mm) position, which allows it to be the only device that can be used either for intraluminal crossing or for subintimal reentry.

## Case-presentation

**Fig. 1:**
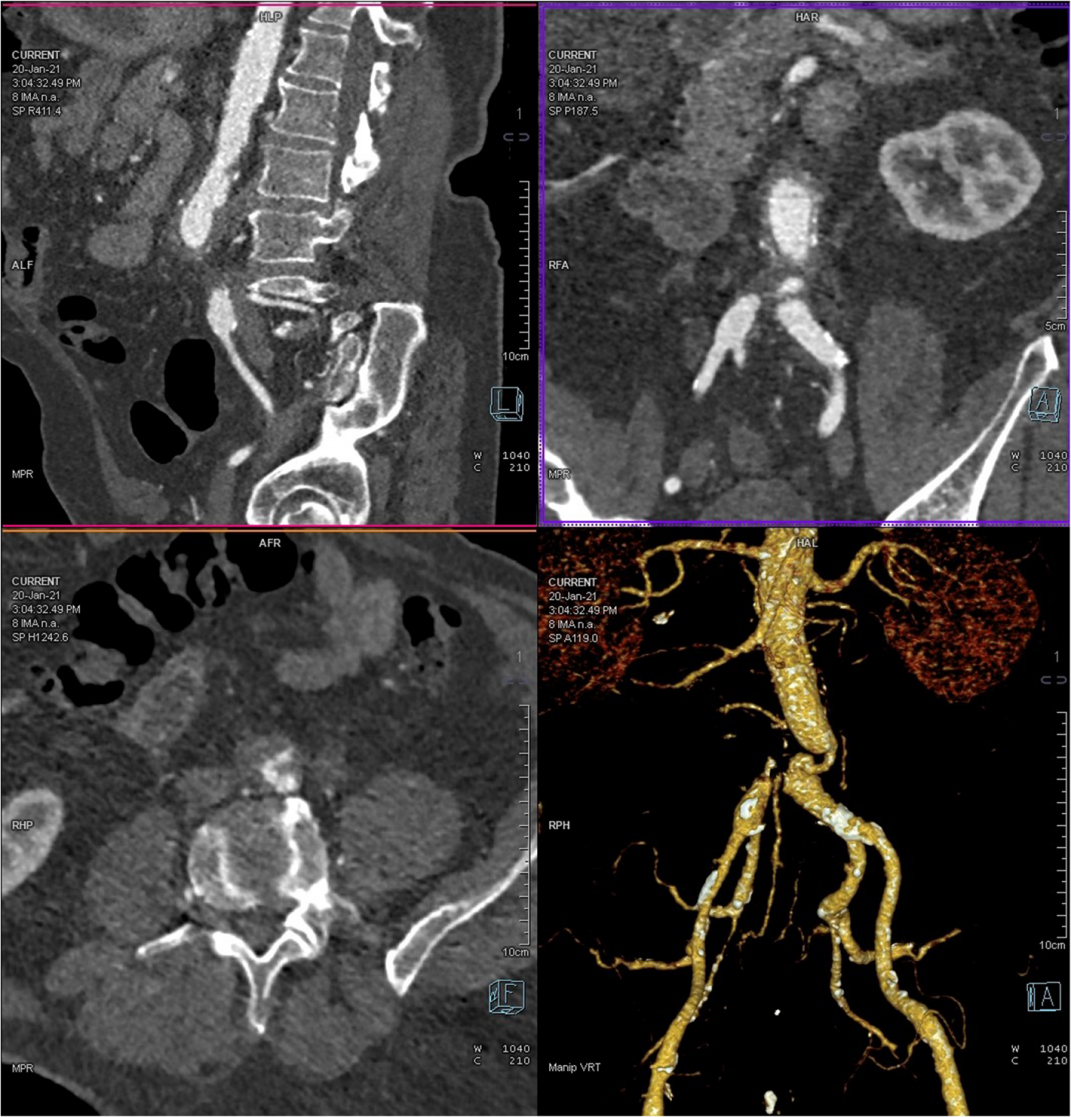
**a** CT angiography images performed before the procedure

A 76-year-old male was admitted for right sural claudication. Aneurysmectomy of the abdominal aorta had been performed 10 years earlier, with an open surgical placement of an aorto-bi iliac dacron graft (18x9mm). The angio-CT scan of the abdomen and lower limbs (Fig. 1) showed that the aorta had a diameter of 25 mm at the distal III, with a mural thrombus, that markedly reduced the flow in the terminal pre-carrefour tract to about 5–6 mm. The left iliac axis presented post-stenotic ectasia up to 16 mm. A 2 cm occlusion of the right common iliac artery was observed, after which the vessel was re-opacified to the distal III due to revascularization by hypertrophic collateral circulation coming from the contralateral hypogastric artery. The right common femoral artery was patent and presented a 7-mm-thick thrombotic apposition. The deep and superficial femoral artery were patent, without non-conditioning atheromasia or significant morphological stenosis. On the left, there was regular patency of the superficial femoral artery and deep common femoral artery.

**Fig. 2:**
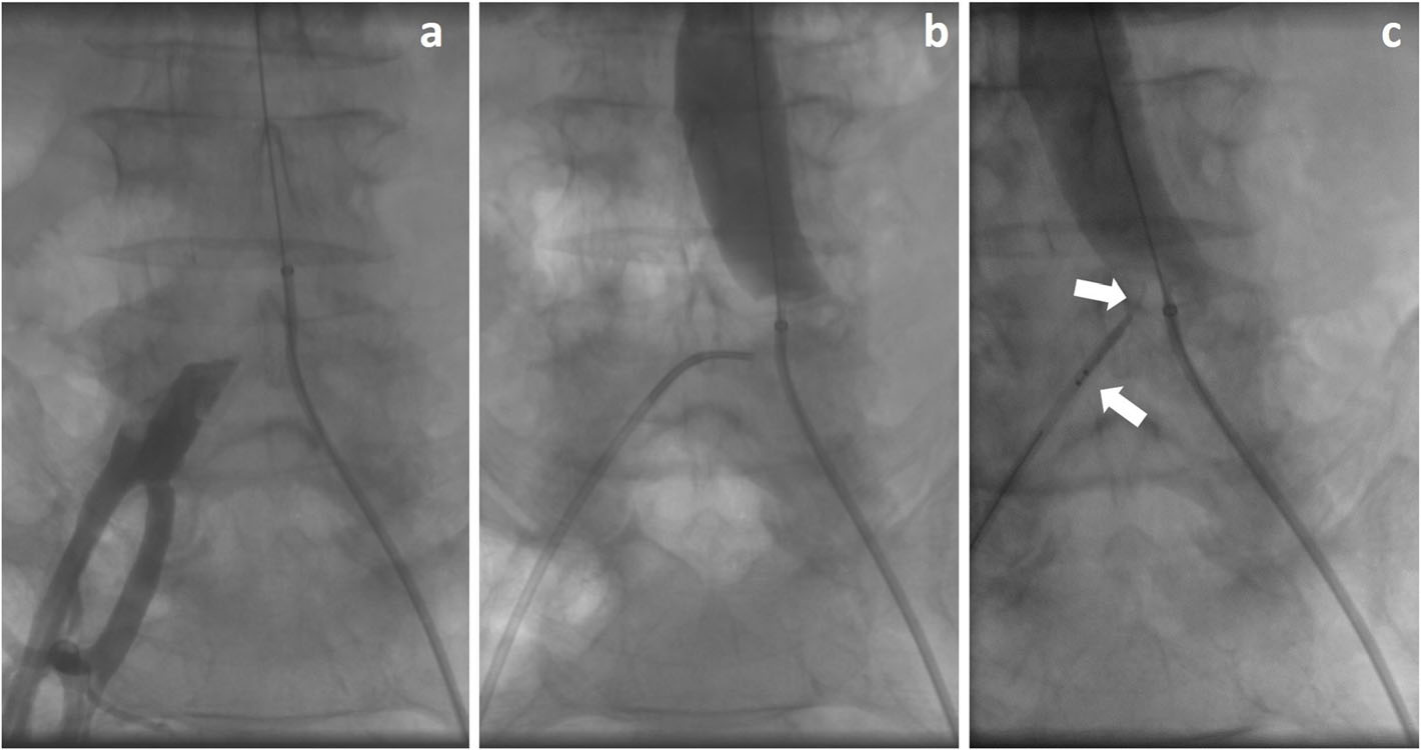
**a** Catheter-based angiography showing occlusion of the right common iliac artery. **b** Catheter-based angiography showing occlusion of the aortic carrefour. **c** Orientation of the GoBack needle using the radio-opaque marker

**Fig. 3:**
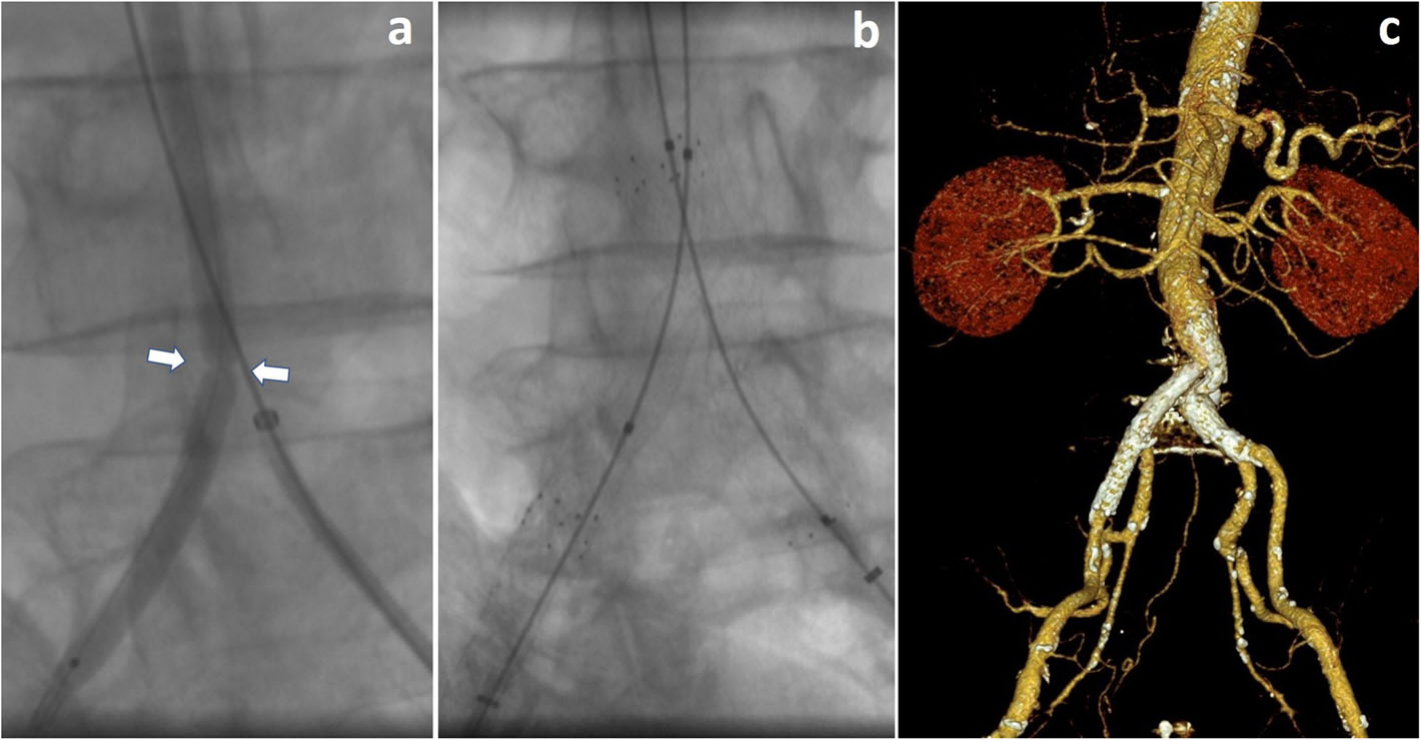
**a** Dilation with a low-profile ballon. Note the resistance to expansion in proximity to the passage created by the GoBack needle (arrows). **b** Deployment of aortoiliac kissing stents. **c** CT-scan follow up at 1 month

It was decided to proceed with a bilateral common femoral arterial approach, with a 6 Fr introducer sheath on the left side and 8 Fr introducer sheath on the right side, after local anesthesia and systemic heparinization (5000 UI). Preliminary angiography via the left common femoral artery confirmed and highlighted the occlusion of the aortic carrefour (Fig. 2a, b). The occlusion of the left common iliac artery was passed using a 5 Fr catheter and 0.035”guidewire. After several unsuccessful attempts were made to cross the occlusion of the right common iliac artery due to lumen obstruction by folds of fabric from the collapsed graft wall. The GoBack catheter 4 Fr with 0.018″ guidewire ASAHI Gladius MG 18 PV ES (Asahi Intecc, Nagoya - Japan), was deployed to create a lumen through the graft fabric, inserted from the right common femoral artery and advanced to the desired location. The needle was then extended to 5–7 mm and aimed and pushed. The radio-opaque marker on the needle’s distal section provides guidance as to the needle tip axial and radial position so the GoBack needle was precisely oriented to easily cross the occlusion (Fig. 2c). Thereafter, a 6 Fr guiding catheter was inserted and pre-dilation was performed (Fig. 3a) with the low-profile Passeo-35 Xeo balloon catheter (BIOTRONIK AG, Bülach - Switzerland) to 4 mm and 5 mm, and then subsequently inflated to 8 mm. Aortoiliac kissing stents were then deployed (Fig. 3b). On the right side, Covera™ Vascular (BD, Franklin Lakes – USA) covered stent (10 × 60 mm) was placed imbricated with an Astron (BIOTRONIK AG) bare-metal stent (10 × 40 mm), with distal landing in the external ipsilateral iliac artery. On the left side, an Epic™ (Boston Scientific Corporation, Natick - USA) vascular self-expanding stent (10 × 60 mm) was inserted. Post-dilation was performed at 10 mm. The final angiographic assessment showed resolution of the occlusion, restored direct flow and increased distal flow, without any complications. Previous sural claudication was resolved andCT-scan follow up at 1 month and DUS follow up at 3 months confirmed the patency of ilio-femoral axis with triphasic flow (Fig. 3c).

## Conclusions

Classic endovascular approaches often fail to cross occlusions, as in this particular case. In the presented case, following failed crossing attempts, the occlusion could have been addressed with stenting of the left iliac axis and a femorofemoral cross-over surgical bypass from the left to right side or conservative management. But, with the assistance of the GoBack catheter, flow was regenerated and stenting was successfully performed in a less invasive, precise and time-efficient manner. The GoBack catheter is an effective vessel lumen crossing and re-entry device, particularly suitable for tough occlusions and interventional radiologists as well as clinicians should consider this catheter as a potential complementary interventional instrumentation tool.

## Data Availability

Data sharing is not applicable to this article as no datasets were generated or analysed during the current study.
